# The State and Tuberculosis

**Published:** 1907-05

**Authors:** 


### THE STATE AND TUBERCULOSIS.

---

G. W. Holden, in discussing the duty of the State in the prevention and control of tuberculosis considers compulsory registration imperative, the records, however, to be accessible only to authorized officials. In case of indigent patients, not under care of a reputable physician, isolation may be necessary. Monthly bulletins should be issued and sent to every practicing physician by the State Board of Health, and in these the subject of tuberculosis should be a prominent feature. Dry street sweeping should be condemned as dangerous, and if the streets can not be properly flushed they should be sprinkled before swept and the sweepings carted away. Anti-spitting laws should be enforced and infected apartments should be disinfected after death. Overcrowding in tenement houses should be abolished so far as possible. Public conveyances, including railway and street cars, should be under sanitary control and the smoke nuisance should be abated. Dairies and herds should be frequently inspected and proper sanitary methods ensured, and there should be more careful inspection of slaughtered animals for home use. The exclusion of consumptive children should be insured by law, and school rooms never used for public meetings or be disinfected after each time of such use. State or city charges, if tuberculosis, should be isolated, and Holden suggests that every general hospital should isolate tuberculous patients regardless of the disease for which they are in hospital. The matter of city and state sanatoria is a complicated one. The possible interference of politics, the expense, size, etc., all have to be considered. Holden advocates county asylums with State aid under the supervision of the State Board of Health as a possible solution.—Journal A. M. A., January 19, 1907.

